# Evaluation of Graft Uptake and Hearing Assessment after Palisade Myringoplasty

**DOI:** 10.31729/jnma.3699

**Published:** 2018-08-31

**Authors:** Poonam KC

**Affiliations:** 1 International Nepal Fellowship-Green Pastures Hospital, Pokhara, Nepal

**Keywords:** *cartilage palisades*, *chronic otitis media*, *large perforation*, *myringoplasty*

## Abstract

**Introduction:**

Cartilage as a graft for closure of tympanic membrane has got superior benefits than other usual grafts (temporalis fascia and perichondrium). Cartilage supported myringoplasty with palisade technique has good result of graft uptake rate, even under difficult conditions. This technique brings very good functional and better long-term results. This study is done to assess graft uptake rate and hearing improvement after myringoplasty with cartilage palisade technique.

**Methods:**

It is a descriptive, hospital based observational study done at Manipal Teaching Hospital, Pokhara between 2014–2017. A total of 45 patients aged between 13 years and 44 years diagnosed with chronic otitis media-mucosal were taken. Pure tone audiometry was done before and six months after surgery. Graft uptake and Post-operative hearing gain was evaluated after six months. Statistical analysis was done by Statistical Package for Social Sciences version 16.0. Statistical significance was

set at P<0.05.

**Results:**

Graft uptake rate was 41 (91.1%). The mean pre-and post-operative pure tone average were 26.88dB and 8.44dB respectively. The post-operative hearing gain was 18.36dB. Hearing improvement after surgery was found to be statistically highly significant with P<0.001.

**Conclusions:**

Cartilage supported myringoplasty using palisade technique is preferred for chronic otitis media-mucosal with large and sub-total tympanic membrane perforation.

## INTRODUCTION

Perforation in the Tympanic Membrane (TM) is deemed to be chronic if present for three months.^1^ Chronic Suppurative Otitis Media (CSOM) is defined by otorrhoea of at least six weeks duration in the presence of a chronic tympanic membrane perforation.^1^ Palisade technique is the most common technique used during cartilage tympanoplasty.^2^ The cartilage graft is cut into several slices or strips, which are subsequently pieced together, medial to the malleus to reconstruct the tympanic membrane.^3^

Persistent perforation of the tympanic membrane causes recurrent ear discharge and hearing loss of varying degrees. Cartilage supported myringoplasty with palisade technique has good result of graftuptake rate ranging from 86 to 100%.^4,5^ This technique brings very good functional and better long-term results.^3^

The study is carried out in Chronic Otitis Media (COM)-mucosal to reconstruct tympanic membrane perforation in order to assess graft uptake result after palisade myringoplasty and hearing improvement post-operatively.

## METHODS

This is a descriptive, hospital based observational study done at ENT department of Manipal Teaching Hospital, Pokhara, Nepal between 2014–2017. Patients aged between 15–45 years were included. Ethical approval was taken from Institutional Review Committee, Manipal Teaching Hospital and written informed consent was taken from individual patient. Inclusion criteria were Chronic Suppurative Otitis Media, tubotympanic type with central perforation, Pure Tone Average (PTA) of the ear to be operated should be between 25 to 45 decibels, ear to be operated should be without discharge for at least 3 months before surgery, no sensorineural hearing loss (adequate cochlear reserve must be present) and operated ear should be the worse hearing ear. Cases excluded were criteria with: 1. Syndrome that can affect middle ear e.g. cleftpalate, Kartagener syndrome, Down syndrome. 2. Malignancy of ear. 3. Concomitant mastoidectomy. 4. History of otologic interventions/ revision myringoplasty 5. Military and recreational professionals. Sample size was calculated using following formula

Sample size (n)


$$\begin{array}{l} \mathrm{Sample}\;\mathrm{size}\;\left(n\right)\\ =\frac{{\text{Z}}^{2}\;\times \text{ P }\times \text{ Q}}{{\text{E}}^{2}} \end{array}$$

P=the graft uptake rate after palisade cartilage myringoplasty = 95%.^6^
Q=100-P,
E=Allowable error= 6.25% of P
Z (Standard deviation)=1.96

With 95% confidence interval, sample size (n) required for the study is 47 cases.

All operative procedures were done under local anesthesia. Permeatal approach was used, except in few cases with post-auricular approach for narrow external auditory canal. Examination under microscope was done. Margin of the tympanic membrane perforation was re-freshened and posterior tympanomeatal flap was elevated. Cartilage was taken from tragus or concha and donor site was closed using small interrupted sutures. Perichondrium was elevated from one side of cartilage using round knife and cut into multiple pieces of appropriate sizes. After the middle ear is packed with gel foam, cartilage graft was placed at first anterior margin, then in parallel to handle of malleus with the perichondrial surface facing laterally. Tympanomeatal flap was repositioned. Patient discharged on next day with 7 days course of oral antibiotics. External auditory canal pack was removed after one week. Pure tone average was done at 3 months and 6 months.

## RESULTS

Total number of patients enrolled for the study was 47. Out of them 2 were lost to follow up and were excluded from the study with remaining 45 patients in study group. Ages of the patients ranged between 13 to 44 years with mean age of 23.44 years.

**Figure 1.**
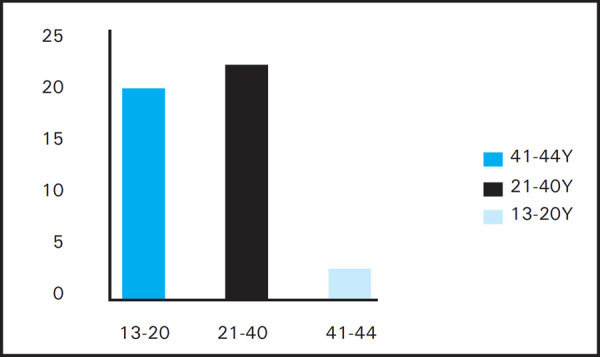
Age Distribution of Patients.

There were 18 (40%) female patients and 27 (60%) male patients. Male: Female ratio was 1.5:1.

**Figure 1.**
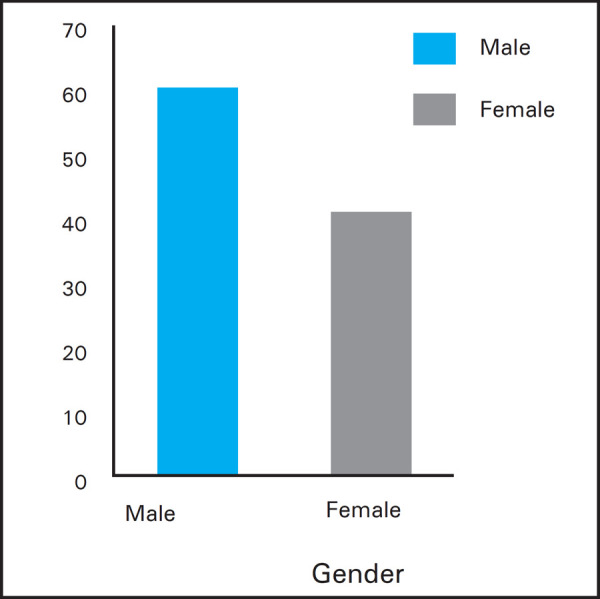
Gender distribution of patients.

Graft uptake was found in 41 patients and residual perforation seen in 4 patients (Table 1).

**Table 1:** Post-operative Graft Status.

	n (%)
Intact	41 (91.1)
Failure	4 (8.9)
Total	45 (100)

For the evaluation and comparision of hearing results, all patients with normal ossicular chain were taken. The mean preoperative PTA was 26.88dB (SD=2.16) and mean post-operative PTA was 8.44dB (SD=4.47). Hearing improvement after surgery was found to be statistically highly significant (P<0.001) (Table 2).

**Table 2:** Comparision of Pre and Post-operative hearing.

	Mean	n	Standard deviation	Standard error mean
Pre-Op	26.88	45	2.16	0.32
Post-Op	8.44	45	4.46	0.66

Paired T-test

P<0.001- statistically highly significant

Mean post-operative hearing gain found as 18.36dB. In relation with TM perforation size, gain was seen more in large perforation than subtotal group (Table 3).

**Table 3:** Post-operative hearing gain.

	n	Minimum	Maximum	Mean	Standard deviation
Gain 45 in PTA	8.3	25	18.36	4.57

## DISCUSSION

This study was done to evaluate the graft uptake rate and post-operative hearing results of myringoplasty using cartilage palisades. Required sample size of this study was 47. However, 45 patients were included as 2 patients were lost during follow-up.

Patient aging from 13 years to 45 years were taken for the study. The mean age of the patients was 23.44 years with minimum age was 13 years and maximum was 44 years. Due to low sample size, no definitive conclusion can be withdrawn with this finding. However, the mean age belong to early adulthood (20s) which is the common age group who undergo myringoplasty procedure as described by other studies.^7,8^

There was no gender preponderance seen for the disease included in the study with male: female ratio = 1.5:1. This signifies that both gender can equally be affected by the COM-mucosal type. After surgery, first assessment was done at 7^th^ post-operative day where suture and External Auditory Canal pack was removed. At 6 weeks, graft uptake was assessed. By this time, gel foam dissolved completely.^9^ The follow-up period in this study ranged from 4 weeks to 6 months. A short follow up period is one of the limitations of this study.

Graft uptake rate after surgery was 91.1%. Postoperative graft uptake and hearing results obtained with this study was comparable to other similar studies. This study shows similar kind of results. Pure tone audiometry was performed in sound treated room with calibrated equipment. Average was calculated from 500Hz, 1 KHz and 2 KHz frequencies. These frequencies were the speech frequencies that keep the importance of subjective hearing. Similar audiometric evaluation was done post-operatively. Post-operative hearing gain was measured from the difference of Postoperative and preoperative PTA value.

In this study, the mean pre-operative AC threshold was 26.88 (SD=2.16) and mean post-operative AC threshold was 8.44 (SD=4.40). Mean post-operative hearing gain was 18.36dB (SD=4.56). The observed difference was statistically highly significant with P<0.001.

Cagdas K, Oral K, Boyraz I^10^ in 2007 performed type 1 tympanoplasty for management of subtotal perforation in 22 patients with palisade cartilage. They found graft acceptance as 95.7%, mean pre and post-operative PTA Air Bone Gradient (ABG) as 31.4±10.7dB and 22.4± 12.0dB respectively.

Vashishtha A, et al^11^ in 2014, performed cartilage palisade in type1 tympanoplasty in 30 patients found graft acceptance by 90% and hearing improved in ABG by 73.57%. Mean pre ABG and post ABG were 29± 6.21dB and 7.33± 3.88dB respectively.

Ozbek C, Ciftoi O, Tuna E, Yazkan O^12^ performed Type 1 tympanoplasty with palisade cartilage in 21 children, found graft uptake rate 100% and hearing improvement as- Mean pre and post PTA-ABG was 25.04dB and 10.33dB respectively.

Cabra J, Monux A^5^ in 2010 performed cartilage palisade tympanoplasty in 64 patients, found graft uptake rate 82.26% and hearing improvement as- Mean pre and post ABG was 25.7dB and 11.25dB respectively.

Shishegar M, Faramarzi A, Tarashi A^13^ performed palisade cartilage tympanoplasty in 27 patients and found Graft uptake rate 100%.

## CONCLUSIONS

Cartilage supported myringoplasty using palisade technique is preferred for chronic otitis media-mucosal with large and subtotal tympanic membrane perforation.
